# RNA Pol II-based regulations of chromosome folding

**DOI:** 10.1016/j.xgen.2025.100970

**Published:** 2025-08-08

**Authors:** Christophe Chapard, Nathalie Bastié, Axel Cournac, Laura Chaptal, Henri Mboumba, Sophie Queille, Agnes Thierry, Olivier Gadal, Armelle Lengronne, Romain Koszul, Frédéric Beckouët

**Affiliations:** 1 Institut Pasteur, CNRS UMR 3525, Université Paris Cité, Unité Régulation Spatiale des Génomes, 75015 Paris, France; 2 Molecular, Cellular and Developmental Biology Department (MCD), Centre de Biologie Intégrative (CBI), Université de Toulouse, CNRS, UPS, 31062 Toulouse, France; 3 Institut de Génétique Humaine, Université de Montpellier, CNRS, Equipe labélisée Ligue contre le Cancer, Montpellier, France

**Keywords:** genome organization, RNA pol II transcription, cohesin, SMC, DNA loop, DNA domain, *S. cerevisiae*

## Abstract

The spatial organization of eukaryotic genomes and its dynamics are of functional importance for gene expression, DNA replication, and segregation. Structural maintenance of chromosome (SMC) complexes are essential instruments of chromosome folding, enabling long-distance intra-chromatid DNA loops. The interplay between these processes is complex. For instance, cohesin, in addition to tethering sister chromatids, dynamically regulates gene expression in mammals by promoting interactions between distal regulatory elements and promoters, whereas transcription itself affects genome folding in many ways. Here, we comprehensively dissect the relative contributions of transcription and cohesin complexes, as well as their interplay, to yeast *S. cerevisiae* genome organization. In particular, we show that transcription (1) is not a motor required to push cohesin during DNA loop expansion, (2) specifically induces the appearance of DNA loops independently of SMC complexes, and (3) interferes with cohesin-mediated DNA loop expansion during their establishment.

## Highlights

• RNA polymerase is not the molecular motor extending the cohesin-dependent loop
• Actively transcribed genes organize the genome independently of SMC complexes
• RNA polymerases act as a barrier for loop expansion
• RNA polymerases are moving barriers

## Graphical abstract

**Figure:**
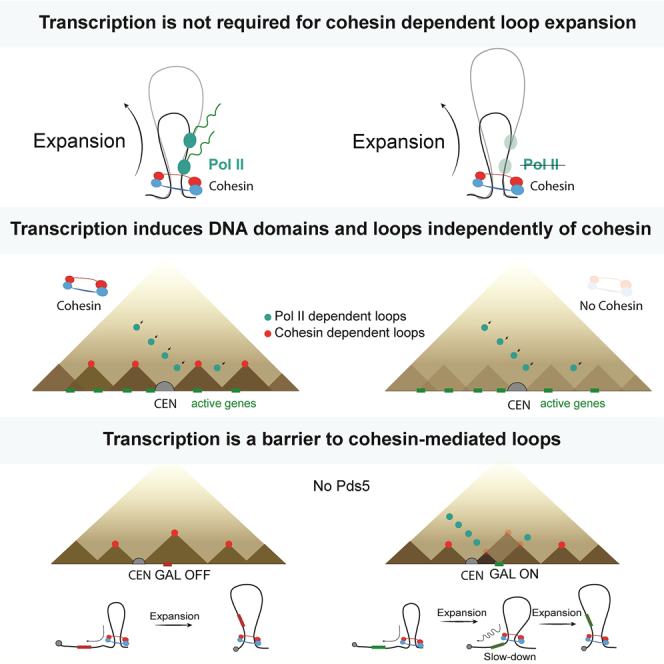

## Teaser

How transcription and cohesin complexes interact to regulate 3D genome organization remains unclear. Chapard et al. dissect their respective contributions and their interplay in shaping the genome architecture of *S. cerevisiae*. The authors reveal that RNA polymerase II is not required for cohesin-dependent loop expansion. Instead, it promotes loop formation independently of SMC complexes and acts as a barrier to cohesin-driven loop expansion.

## Introduction

Chromosomes exhibit a three-dimensional (3D) organization that can influence or regulate DNA processes, including gene regulation, DNA repair, or segregation.^1^^,^^2^^,^^3^ This organization consists of an intertwined network of structures such as long-range DNA loops, large self-interacting chromosomal domains of up to hundreds of kb, and chromosome compartments.^4^^,^^5^^,^^6^ These layers of organization are influenced, or regulated, by molecular processes involving, for instance, topoisomerases, transcriptional machineries, or the ubiquitous structural maintenance of chromosome (SMC) complexes (cohesin, condensin, and Smc5/6).^7^^,^^8^^,^^9^^,^^10^^,^^11^^,^^12^

The loop extrusion (LE) model proposes that SMC rings organize genomes by gradually enlarging small DNA loops into larger structures until they encounter a roadblock and/or are removed from DNA by the releasing factor Wpl1.^4^^,^^13^^,^^14^^,^^15^^,^^16^ CCCTC-binding factor (CTCF) has been identified as a major roadblock to cohesin-mediated loop expansion in mammals,^3^^,^^11^^,^^17^ while other roadblocks have also been described in mammals and in organisms lacking CTCF, such as in yeast.^18^^,^^19^^,^^20^^,^^21^

The findings that cohesin localizes between genes that are transcribed in a convergent orientation in organisms as different as *Saccharomyces cerevisiae* and *Schizosaccharomyces pombe* and in mammals lacking both CTCF and Wpl1 suggest that active transcription also displaces and/or prevents translocation of cohesin along DNA and/or interferes with the cohesin-loading process.^22^^,^^23^^,^^24^^,^^25^^,^^26^^,^^27^ It was, for instance, suggested that transcription may push cohesin rings during DNA loop expansion.^28^ Other studies suggest that transcription negatively regulates cohesin-dependent DNA loops^24^^,^^29^^,^^30^^,^^31^^,^^32^ or is dispensable for the accumulation of cohesin along non-transcribed bacterial DNA introduced in yeast.^33^ Additionally, CTCF/cohesin-independent enhancer-promoter contacts, which may require RNA polymerase II (RNA Pol II) for their formation, have also been described in mammals.^10^^,^^12^^,^^29^^,^^34^ Overall, these studies highlight roles for active transcription and cohesin-mediated loop expansion in eukaryote functional genome organization, but the interplay between these processes remains unclear and varies from species to species.

In the present study, we show that in *S. cerevisiae*, RNA Pol II is not required for cohesin-mediated DNA loop expansion but nevertheless directly impacts chromosome 3D folding by promoting long DNA loops independently of SMC complexes. These RNA Pol II-dependent loops bridge, for instance, a highly expressed gene to active neighboring genes in *cis*. Furthermore, we show that active transcription, which results in the formation of transcription-induced domains (TIDs), antagonizes the establishment of cohesin-dependent loops but has little impact on their maintenance once these structures are formed. Finally, we demonstrate that transcribed regions act as semi-permissive barriers to dynamic cohesin-mediated DNA loop expansion. Altogether, these results reveal the respective contributions of two ubiquitous players of chromosome metabolism, transcription and the SMC cohesin, in shaping the chromosome into two distinct types of chromatin loops.

## Results

### RNA Pol II is not essential to expand DNA loop

**Figure 1:**
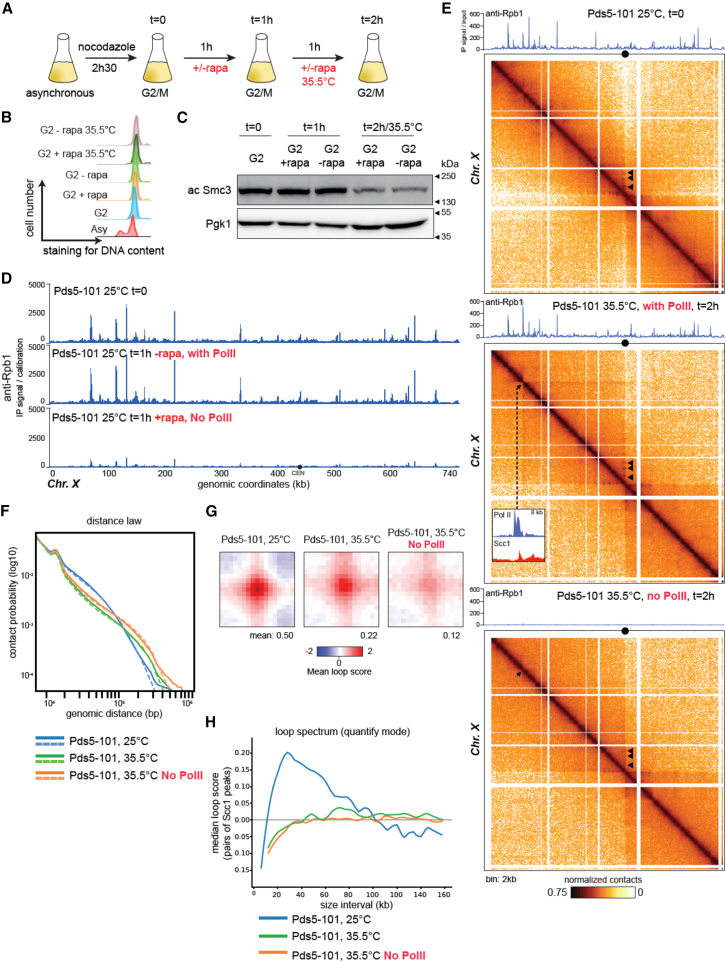
Expansion of cohesin-dependent loop occurs in the absence of RNA Pol II (A) Experimental protocol used to process synchronized yeast cultures. Cells (PP6494) expressing Pds5-105 and the anchor-away system were arrested in G2/M using nocodazole, treated or not with rapamycin for 1 h to remove the Rpb1-FRB and Rpb3-FRB subunits of RNA Pol II from the chromosomes, and then shifted at a restrictive temperature (35.5°C) to inactivate Pds5-101. (B) Cell synchronization monitored by flow cytometry. (C) Western blot assessing the loss of Smc3 acetylation induced by inactivation of Pds5-101 at 35.5°C. Pgk1: loading control. (D) Calibrated ChIP-seq profiles showing the distribution of Rpb1 (RNA Pol II) after and before rapamycin (i.e., no RNA Pol II and 1 h) treatment (STAR Methods) at permissive temperature. (E) Hi-C contact maps (bin: 2 kb) and corresponding RNA Pol II ChIP-seq profiles (anti-rpb1, atop maps) of chromosome X for *pds5-101* cells at permissive (25°C) or restrictive (35°C) temperature with or without transcription. Black arrows point at CEN loops. On the middle map, a dotted black line points at the base of the stripe-like structure, accompanied by a magnification centered on the base and highlighting the corresponding local RNA Pol II and cohesin distribution assessed by the ChIP-seq experiment. (F) Contact probability curves P(s) representing the average contact frequency as a function of genomic distance (bp). The dotted lines represent independent biological replicates. (G) Mean profile heatmap of cohesin loops using the quantify mode of Chromosight^37^ in the presence or absence of transcription (maps in E). Loops were called by Chromosight on the *pds5-101* Hi-C map at permissive temperature (25°C) and computed using the pairs of peaks of cohesin for the three datasets. (H) Loop spectrum using the quantify mode of Chromosight. The loop spectrum shows the loop score computed by Chromosight as a function of the genomic distance separating pairs of peaks of cohesin for the three datasets.

The nature of the molecular motor responsible for cohesin-dependent DNA loop expansion has been debated for years. One model proposes that transcribing polymerases drive or contribute to loop expansion by pushing cohesin along the DNA, while another states that DNA loop expansion is stimulated by energy emanating from the ATPase heads of cohesin. In the absence of Pds5, an essential ancillary cohesin factor,^35^ sister-chromatid cohesion is lost, and intra-chromatid DNA loops extend over much longer distances.^36^ If RNA Pol II is the molecular motor needed for the expansion of DNA loops, then the depletion of RNA Pol II from the chromosomes should prevent, or at least attenuate, the expansion resulting from Pds5 inhibition. To test this, we used a yeast strain expressing a thermo-sensitive allele of Pds5 (*pds5-101*^35^) and carrying the anchor-away system to induce the depletion of RNA Pol II subunits (Rpb1 and Rpb3) from chromosomes in a controlled manner using rapamycin (STAR Methods). An overnight culture was synchronized in G2 using nocodazole, and half of it was then treated with rapamycin to inhibit RNA Pol II transcription (Figures 1A and 1B). After 1 h, both treated and untreated cultures were shifted to restrictive temperatures to inactivate Pds5 (Figures 1A and 1B). The loss of Smc3 acetylation, visualized by western blot, confirmed the efficacy of Pds5 inactivation (Figure 1C). A calibrated chromatin immunoprecipitation sequencing (ChIP-seq) experiment confirmed that RNA Pol II occupancy was greatly reduced after 1 h of rapamycin treatment (Figure 1D) and over the course of Pds5 depletion (Figure 1E). As expected,^36^ derived from Hi-C, a proximity ligation method that captures the organizational structure of chromatin in three dimensions, contact maps and the contact decay curve representing the contact frequency as a function of genomic distance P(s) show that inactivation of Pds5-101 in non-depleted RNA Pol II cells leads to the spreading of intrachromosomal contacts over longer distances, engages centromeres in a series of large loops (centromere loops or CEN loops), and induces the appearance of large DNA domains along the chromosomal arms (Figures 1E–1G). These domains are delimited by stripe-like structures, which result from the engagement of a discrete locus RNA Pol II rich in a series of hotspots of intrachromosomal contacts (e.g., Figures 1E, middle inset, and S1A). Loop calling using Chromosight^37^ shows that the number of loops with anchors at cohesin peaks is roughly similar in cells with and without Pds5 inactivation (322 at 25°C vs. 256 at 35.5°C, respectively). However, the mean loop scores (MLSs) for these “cohesin loops” (STAR Methods) show a strong reduction at restrictive vs. permissive temperatures (0.50 vs. 0.22, respectively) (Figures 1G, S1B, and S1C). Pairs of cohesin-enriched sites close to each other display fewer contacts upon Pds5-101 inactivation (Figure 1H; STAR Methods), while sites separated by longer distances now display enriched contacts compared to the wild type (WT) (Figure 1H).

The long-range dotted pattern observed in Hi-C contact maps and P(s) curves of *pds5-101* mutants was strongly altered upon the inhibition of RNA Pol II-mediated transcription (Figure 1E, bottom map). First, in the absence of RNA Pol II, loop expansion processed over even longer distances along the chromosomes, as shown by the P(s) curve (Figure 1F, orange curve). In addition, the absence of RNA Pol II also induces the loss or a strong attenuation of the remaining discrete contact hotspots retained along the chromosome arms in the absence of Pds5 (Figures 1E, black triangles, and 1G). Consequently, in RNA Pol II-depleted cells, the centromere emerges as the only locus determining a loop base, resulting in a contact stripe extending over the arm with no specific enrichment hotspots. Altogether, these results show that although active transcription influences the positioning of the loop base, it is not essential to expand them.

### Transcription mediates DNA structures independently of SMC complexes

**Figure 2:**
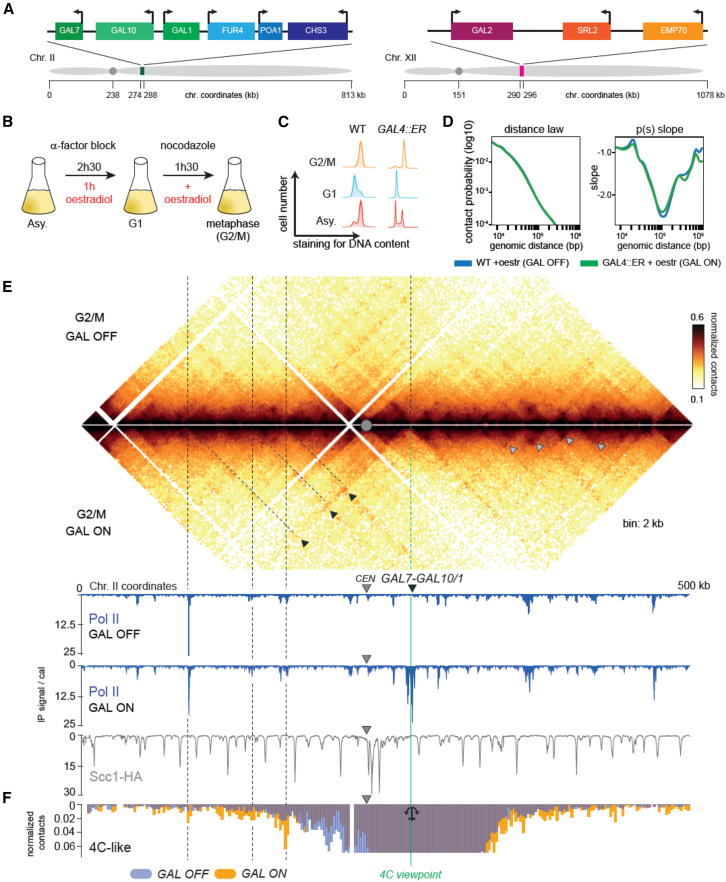
Activation of *GAL* genes delimits chromatin domains and induces discrete long-range DNA loops (A) Schematic representation of *GAL* loci on chr. 2 and 12. (B) Schematic representation of the experimental procedure used to active *GAL* genes in synchronized cultures. (C) Cell synchronization monitored by flow cytometry. (D) Left: contact probability curves P(s) representing the average contact frequency as a function of genomic distance (bp). Right: corresponding local derivative curves. (E) Hi-C contact maps (bin: 2 kb) and corresponding RNA Pol II ChIP-seq profiles of a section of chr. 2 containing the *GAL7-10/1* locus in cells synchronized in G_2_ without (top, W303-1A) or with (bottom, BEN15) estradiol. Gray arrowheads point at cohesin-associated loops. Loops with the *GAL7-10/1* locus as one base are indicated with black triangles. For each loop, the coordinate of the other base is identified following the dark dotted lines. The RNA Pol II ChIP-seq profile in the presence of estradiol and the cohesin (Scc1) ChIP-seq profile of G2-arrested cells are shown in dark blue and gray, respectively (strains: FB218-4a, FB218-8d, and yNB30.2-14b). (F) Virtual 4C analysis using a viewpoint (anchor) just upstream the GAL7-10/1 locus. The dark purple profile shows enriched contacts when the GAL locus is activated. The RNA Pol II ChIP-seq profile in presence of estradiol and the cohesin (Scc1) ChIP-seq profile of G2-arrested cells are shown in dark blue and gray, respectively (strains: FB218-4a, FB218-8d, and yNB30.2-14b).

To further explore the effect of transcription on loop establishment and positioning, we investigated the consequence of galactose-inducible activation of the well-studied *GAL7-GAL10/GAL1* locus (*GAL7-10/1*, GAL or GALactose metabolism) on *S. cerevisiae* chr. 2 (Figure 2A). We used a strain expressing the *GAL4-ER-VP16* fusion protein that allows the inducible activation of *GAL* promoters in response to estradiol, even in the presence of glucose^38^ (STAR Methods). The direct comparison of cells grown in identical culture conditions is therefore allowed, as opposed to glucose- vs. galactose-treated cells (Figures S1D and S1E). Cells growing exponentially in glucose medium were arrested in G1 using α factor, and *GAL* genes were then induced using estradiol (STAR Methods). The cells were next released from G1 and allowed to progress into the S phase before being arrested in G2/M with nocodazole (Figures 2B and 2C). Hi-C contact maps and Scc1 and RNA Pol II ChIP-seq data were generated for the different time points in the presence or absence of estradiol induction of GAL genes. The estradiol treatment did not affect the global chromosome compaction, as shown by the identical P(s) of treated and untreated cells (Figure 2D). As expected, upon estradiol treatment, activated *GAL* loci were highly enriched in RNA Pol II compared to untreated cells (Figure 2E). Both the ratio of induced vs. uninduced maps (Figure S1D) and comparison of the normalized maps (Figure 2E) underly the changes accompanying *GAL7-10/1* gene induction. Firstly, transcription of the highly expressed locus is associated with a strong limitation of *cis*-DNA contacts across both sides of the locus, hence translating as a “border” in a contact map (Figures 2E, dotted green line, and S1D). In addition, a dotted contact pattern appears at the level of the induced *GAL7-10/1* genes, pointing to the formation of long-range chromatin contacts that bridge the transcribed locus with neighboring regions (Figure 2E). These loop-like structures differ from G2/M cohesin-mediated loops as follows: (1) they are insensitive to the presence of a centromere and can bridge regions on both chromosome arms, a stark difference from cohesin-mediated DNA loops, for which the centromeres represent impassable boundaries,^18^^,^^19^^,^^39^ and (2) their bases do not overlap with cohesin-enriched positions (Figure 2E). A virtual 4C (unbiased detection of all genomic regions that interact with a particular region of interest) representation shows that these loops bridge the induced GAL locus with RNA Pol II-enriched regions up to 100 kb away (Figure 2F).

**Figure 3:**
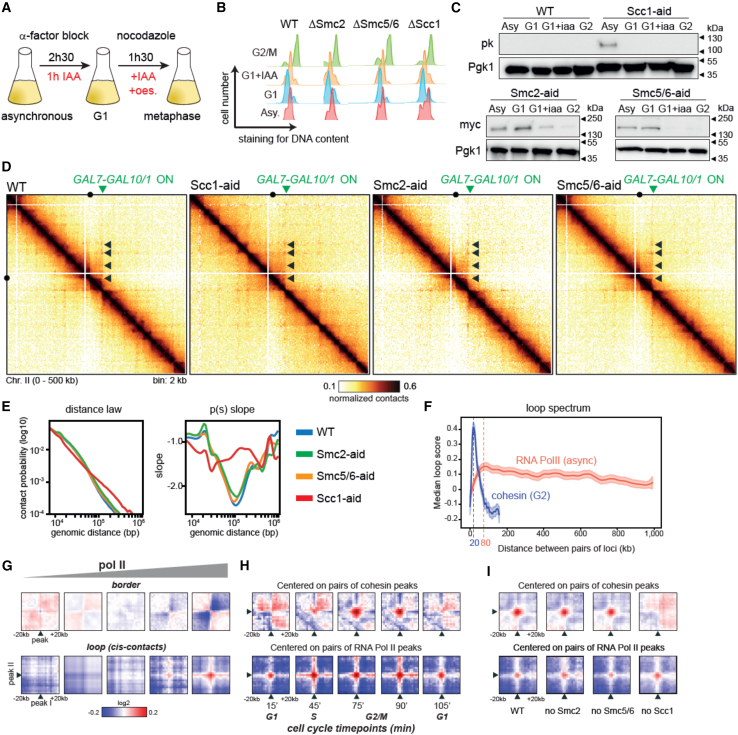
Active RNA Pol II induces discrete long-range DNA loops independently of SMC complexes (A) Schematic representative of the protocol used to active *GAL* genes in synchronized cultures depleted for SMCs. (B) Cell synchronization monitored by flow cytometry. (C) Western blotting assessing SMCs depletion (strains FB220-1b, FB219-2a, FB242-8c, and yCH001-9a). (D) Hi-C contact maps (bin: 2 kb) of the chromosome 2 region containing the activated GAL7-10/1 locus (green arrow) following depletion of SMCs by IAA (indole-3-acetic acid or auxin) addition. Dotted contacts by the GAL loci are underlined by black arrowheads. (E) Left: contact probability curves P(s) representing the average contact frequency as a function of genomic distance (bp). Right: corresponding local derivative curves. (F) Loop spectrum showing the loop score computed by Chromosight as a function of the genomic distance separating 2 RNA Pol II peaks. (G) Pileups of RNA Pol II peaks at the positions sorted by RNA Pol II levels (STAR Methods) along the diagonal (top) and at long distances (60–460 kb) (bottom).^40^ (H) Pileup plot for cohesin loops (pairs of Scc1 peaks separated by genomic distances between 10 and 50 kb) and for RNA Pol II loops (pairs of RNA Pol II peaks at long distances [60–460 kb]) at different time points during the mitotic cell cycle. (I) Pileup plot for RNA Pol II-enriched regions over long distances (60–460 kb) and for cohesin loops for WT and SMC-depleted cells (same yeast strains as in C).

These RNA Pol II loops are not specific to G2 and were also observed in G1 synchronized cells following GAL induction (Figures S1F and S1G), further suggesting that cohesin is not involved in their formation, as confirmed by their persistence in cells depleted for Scc1 (Figures 3A–3E). The long DNA loops at the activated GAL locus are also independent of the other SMC complexes, condensin and SMC5/6, as shown by the controlled degradations of either auxin-inducible degron (AID) tagged Smc2-AID (condensin) or Smc5-AID/Smc6-AID in cells expressing GAL genes (Figures 3A–3E).

We then explored genome wide whether transcribed genes display enriched contacts between each other. To do so, we generated a loop spectrum by computing loop scores for pairs of loci using Chromosight “quantify” mode and plotting them as a function of the genomic distance between them (see STAR Methods).^37^ First, we computed the loop spectrum using pairs of loci in *cis* enriched in cohesins in G2 (Figure 3F, blue curve).^18^ The spectrum displays the expected maximum for genomic distances around 20 kb, corresponding to the average cohesin loop sizes at this stage.^18^^,^^19^ When applied on pairs of loci enriched in RNA Pol II in *cis*, the analysis reveals an enrichment in contacts over very long distances, up to hundreds of kb, with a maximum score of around 80 kb (Figure 3F, red curve), showing that active genes have a slight tendency to contact each other in *cis*. To determine further the correlation between gene expression levels and the local apparition of borders and loops, we computed pile-up contact maps of 40 kb windows of asynchronous WT cells^40^ centered on RNA Pol II peaks classified according to their RNA Pol II occupancy levels (STAR Methods; Figure 3G). The transcription-associated borders are more marked when the sites are highly enriched in RNA Pol II, which is in line with previous observations (Figure 3G, top).^30^^,^^41^^,^^42^ The loop signal also appears stronger for pairs of highly expressed genes (Figure 3G, bottom), and was strongly reduced in RNA Pol II-depleted cells (Figure S1H). It should be noted that the RNA Pol II depletion condition does not appear to strongly affect the overall positioning and strength of cohesin loops on the genome, which is in contradiction with a recent study (Figure S1H).^28^

The RNA Pol II loop contacts are maintained throughout the cell cycle, in contrast to cohesin-dependent DNA loops, which only form during the S and G2/M phases (Figure 3H). We computed the genome-wide pileup in cells depleted for SMC complexes, showing that the RNA Pol II signal was maintained upon depletion of one or the other SMC complex (Figure 3I). We also observed that inactivation of Pds5-101 reduces the intensity of RNA Pol II loops, indicating that the expansion of cohesin loops may interfere with the establishment or maintenance of RNA Pol II loops (Figure S1C).

Altogether, these results show that transcription activation in budding yeast, besides limiting *cis*-chromosome Hi-C contacts between flanking regions, is responsible for generating long-range *cis*-contact associations between RNA Pol II-occupied regions. Transcription *per se* is therefore directly involved in the 3D structuration of the yeast genome.

### Transcription restricts the formation of cohesin-dependent intrachromosomal contacts

**Figure 4:**
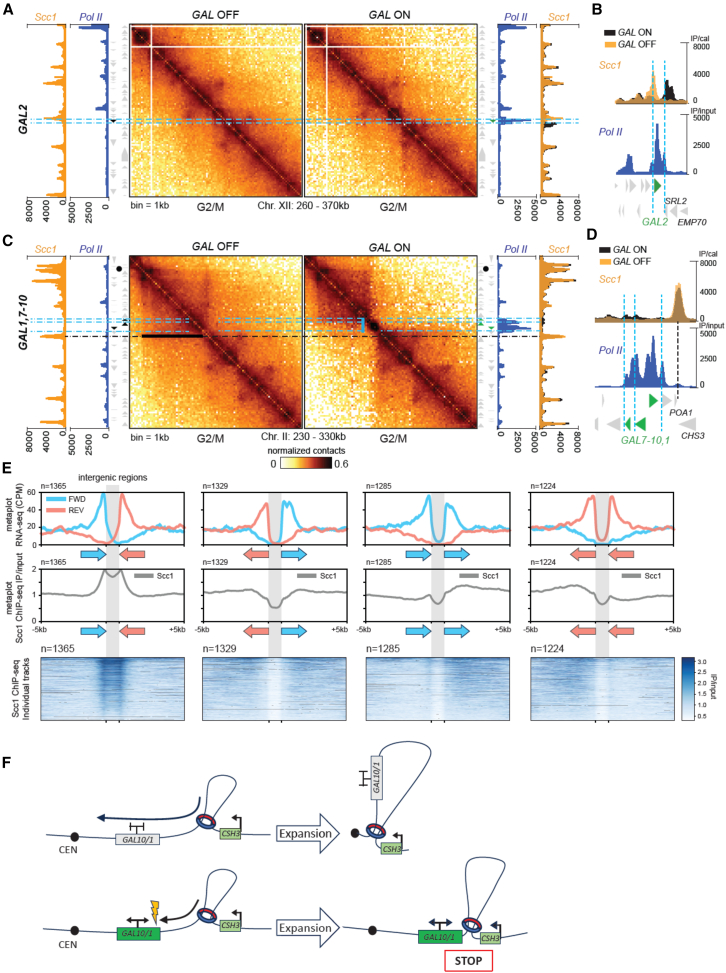
Transcription-dependent DNA borders hinder establishment of cohesin-mediated DNA structures (A) Hi-C contact maps (bin: 1 kb) of a region of chr. 12 containing the *GAL2* locus for strains W303-1A and FB220-1b, with the corresponding RNA Pol II and Scc1 ChIP-seq profiles (strains: FB218-4a and FB218-8D). Cells are synchronized in metaphase, without (left) or with (right) estradiol-induced activation of *GAL2*. Note that Scc1 ChIP-seq profiles before estradiol-mediated induction of *GAL2* are plotted in light orange, while those after induction are in black. Dotted blue lines highlight the position of the displaced cohesin peaks. (B) Top: magnification of the overlaid Scc1 ChIP-seq profiles before (light orange) and after (black) estradiol-mediated induction of *GAL2* on chr. 2. Bottom: corresponding RNA Pol II ChIP-seq profile (blue). (C) Same as (A) but for the region of chr. 2 containing the *GAL7-10/1* locus. The solid black line indicates the stripe-like structure present on the Hi-C map. The black line points to the cohesin peak at the base of the stripe-like structure between POA and CSH3 genes. The vertical blue line highlights the formation of the transcription-induced domain (TID) at the *GAL7-10/1* locus. The dotted blue lines highlight Scc1 peaks on the region upon induction of the *GAL* genes. (D) Same as (B) but for the region containing the *GAL7-10/1* locus. Dotted blue and dark lines are the same as those in (C). (E) Top and middle: metaplot profiles of RNA sequencing (RNA-seq) and Scc1 ChIP-seq signals measured^43^ across intergenic regions ±5 kb, classified into 4 groups according to the orientations of the flanking genes (forward and reverse). Bottom: individual tracks of the Scc1 ChIP-seq profiles. (F) Model illustrating the mechanism by which transcription during G1/S phases interferes with the translocation process, inducing loop expansion.

We then analyzed how the strong activation of the *GAL* genes in G1 influences the formation of cohesin-mediated chromatin loops during the S phase (Figures 4A–4D and S2).^18^^,^^19^ Previous work showed that the galactose-induced transcription of the *GAL2* locus prevented the accumulation of cohesin observed on the gene body in G2/M when cells were grown in glucose.^24^ Instead, cohesin enrichment was observed downstream, at the 3′ end of the active gene.^22^ Hi-C data reveal that cohesin repositioning at this locus also results in the repositioning of the base of the associated DNA loop (Figures 4A, 4B, dotted blue lines, and S2B), leading to a decrease in loop size by about 2 kb. This result demonstrates that active transcription influences the positioning of cohesin-dependent loops during their establishment in the S phase.

In contrast to inactive *GAL2*, the silent *GAL7-10/1* in G2/M-arrested cells is devoid of cohesin enrichment*.* The *GAL7-10/1* locus lies within a region delimited by a stripe that emerges from the closest convergent genes (*POA1* and *CHS3*) 3.8 kb away and extends up to the centromere, 38 kb upstream (Figure 4C, plain and dotted black line). This stripe reflects enriched contact between the *POA1-CHS3* position and all positions up to the centromere. It disappears when Scc1 is depleted and is therefore cohesin dependent (Figure S2A). Estradiol activation of *GAL7-10/1* prior to the S phase, confirmed by a large RNA Pol II enrichment, strongly modifies the contact pattern, with the induction of the 6.2-kb-long RNA Pol II track abrogating the apparition of the stripe pattern (Figures 4C and 4D). Note that the activation of the large GAL gene region also results in the formation of TID signals, i.e., short-range contacts between adjacent DNA segments overlapping RNA Pol II deposition and generating a border in a contact map (Figure 4C, blue line), a likely result of an insulating effect of trains of RNA polymerases observed in bacteria and mammals.^42^^,^^44^

In addition, calibrated ChIP-seq analysis revealed slight but significant cohesin enrichments at the 3′ end of the actively transcribed *GAL7*, *GAL10*, and *GAL1* genes, compared to inactive conditions (Figures 4D and S2C). In contrast to most of the cohesin peaks that appear at sites of convergent transcription,^24^ the enrichments affected both co-oriented (*GAL7*) and divergent (*GAL10-1*) genes. This result reveals that active transcription positions cohesin at the 3′ end independently of the orientation of the neighboring genes. A genome-wide analysis of cohesin enrichment over pairs of genes classified according to their relative chromosomal orientation highlighted that this is a general phenomenon, with a clear tendency for cohesin to accumulate in 3′ positions of colinearly oriented genes, as previously suggested^45^ (Figure 4E).

Altogether, the results of our experiment indicate that transcribing polymerases have an active role in the establishment of cohesin-mediated DNA folding, not only redistributing cohesin locally but also affecting long-range chromatin structuration by abrogating cohesin-mediated structure apparition (see model in Figure 4F).

### Transcription interferes with, but does not prevent, the maintenance of cohesin-dependent DNA structures in G2/M

**Figure 5:**
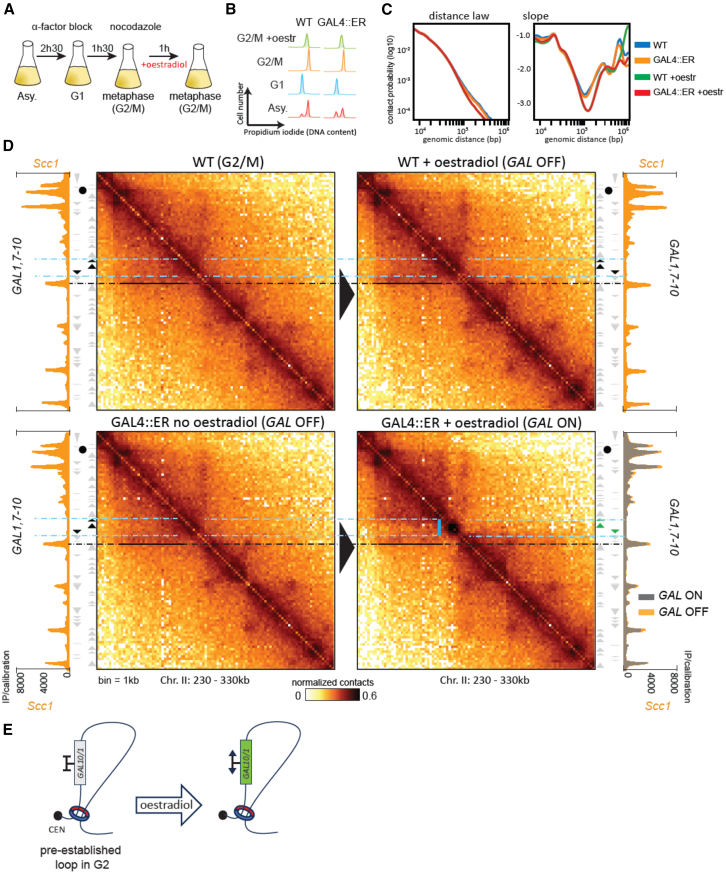
Cohesin-mediated DNA structures are maintained upon the appearance of transcription-dependent DNA borders (A) Schematic representation of the experimental protocol followed for estradiol-induced GAL induction during G2. (B) Cell synchronization was monitored by flow cytometry. Meta, metaphase; Asy, asynchronous; oe, estradiol. (C) Left: contact probability curves P(s) representing the average contact frequency as a function of genomic distance (bp). Right: corresponding local derivative curves. (D) Hi-C contact maps (bin: 1 kb) for strains W303-1A and BEN15 and Scc1 ChIP-seq profiles for strains FB218-4a and FB218-8D, showing the effect of the activation of the *GAL7-GAL10/1* locus during G2 on both chromatin loops and cohesin enrichment sites. Top: control WT cells (W303-1A). Bottom: GAL4-ER-VP16-expressing cells (BEN15) allowing the estradiol-induced activation of *GAL* genes. The solid black line indicates the stripe-like structure present on the Hi-C map, with the dotted black line pointing at its position on the ChIP-seq tracks. The vertical blue line highlights the formation of the transcription-induced domain (TID) at the *GAL7-10/1* locus. The dotted blue lines highlight extremities of the *GAL* genes on the Scc1 ChIP-seq tracks. (E) Model illustrating the absence of an effect of transcription activation on the maintenance of DNA loop in G2/M when the bases of the DNA loop are placed outside the open reading frame (ORF).

To analyze whether and how transcriptional activation interferes with the maintenance of cohesin-mediated WT chromatin loops, we activated transcription *after* the establishment of cohesin-mediated loops during the S phase. Cells were arrested in G2/M, and the *GAL7-10/1* locus was induced by estradiol. Cells were then processed with Hi-C and ChIP-seq (Figures 5A–5D). Hi-C maps revealed that transcriptional activation of *GAL7-10/1* after cohesin-mediated loop establishment results in a TID and the corresponding border but has little, if any, impact on the maintenance of the local 3D structure (Figure 5D). In addition, cohesin deposition at the activated locus also remained unchanged (Figure 5D). These experiments show that while active transcription modulates the formation of cohesin-mediated DNA loops and structures, once established, the latter are largely insensitive to subsequent transcription activation of regions within the structures. This observation argues against an important *de novo* loading and/or highly dynamic translocation of cohesins in G2/M. In contrast, cohesin deposition at the *GAL2* locus was affected upon the activation of transcription in G2/M, as previously described^22^ (Figure S3A). This change was associated with a widening of the associated DNA loop signal as seen on the Hi-C map, possibly due to partial repositioning of the loop anchor in a fraction of the cell population (Figure S3B). Thus, unless activation of transcription occurs immediately at the base of a DNA loop, as is the case for *GAL2*, cohesin-dependent DNA structures and loops are maintained and resistant to the constraints induced by transcription (model in Figures 5E and S3C).

### Transcription is an active and semi-permissive barrier for loop expansion

**Figure 6:**
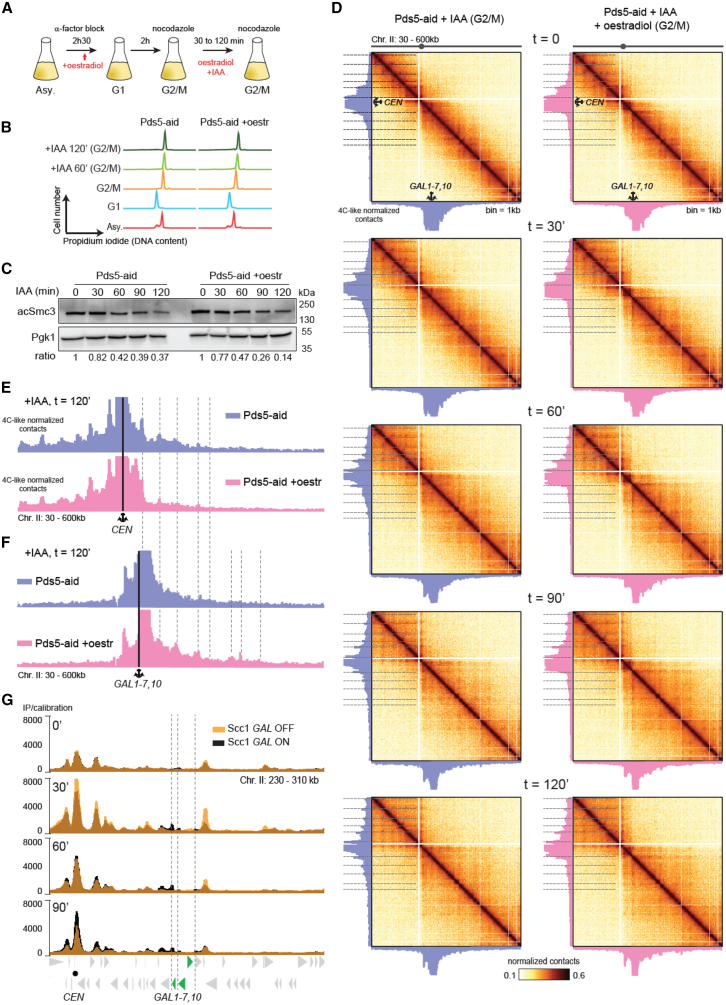
Transcription-dependent DNA borders are semi-permissive barriers to cohesin-mediated DNA loop expansion (A) Schematic representation of the experimental protocol for auxin-mediated degradation of Pds5 in G2 in the presence or absence of estradiol-induced GAL gene activation. (B) Synchronization is controlled with by flow cytometry until 120 min after the addition of IAA. Asy, asynchronous; G1, α factor arrest; Meta., metaphase; oe, estradiol. (C) Pds5 degradation was assessed using Smc3 de-acetylation by western blot. Degradation was quantified relatively to the loading control Pgk1 (ratio). (D) Hi-C contact maps (bin: 2 kb) of a region (30–600 kb) of chr. 2 for FB220-2a and FB220-8c cells during auxin-mediated Pds5 depletion (+IAA). The maps are ordered from top to bottom according to the time in presence of IAA. Time 0: no IAA, and then 30, 60, 90, and 120 min following IAA addition. For each map, two 4C-like profiles using either CEN (*y* axis) or *GAL7-GAL10/1* (*x* axis) loci as anchors are plotted. (E) Magnifications of the 4C-like profiles using CEN as an anchor along chr. 2 in the absence (top, blue) or the presence (bottom, pink) of GAL gene induction, 120 min after IAA-mediated Pds5 depletion. (F) Magnifications of the 4C-like profiles using *GAL7-GAL10/1* as an anchor along chr. 2 in the absence (top, blue) or the presence (bottom pink) of GAL gene induction, 120 min after IAA-mediated Pds5 depletion. (G) Overlaid Scc1 ChIP-seq profiles of a region (230–310 kb) of chr. 2 with (black, strain FB218-4a) or without (yellow, strain FB222-1c) GAL activation and throughout Pds5 depletion (from top to bottom). CEN, centromere. Green arrow: *GAL7-GAL10/1* locus.

**Figure 7:**
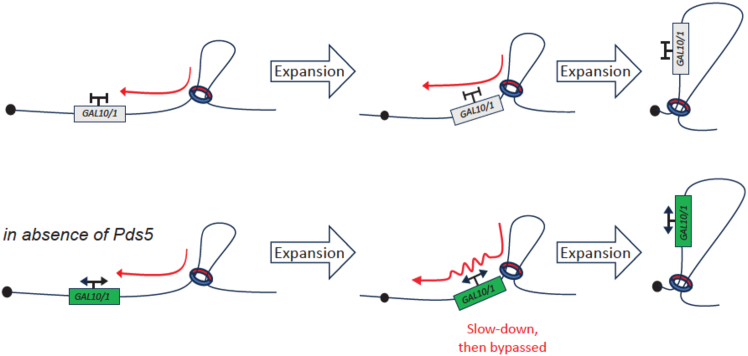
Model explaining how Gal activation slows down the expansion of the long CEN loops induced by Pds5 depletion

Overall, our results show that activation of highly expressed genes (here, *GAL7-10/1*) modulates the formation of loops, either by affecting cohesin loading or by generating a domain conflicting with the translocation of cohesin-extruding DNA loops. To directly explore the impact of transcription on cohesin-mediated looping dynamics, we explored how GAL gene transcription influences the kinetics of CEN loop formation induced by Pds5 inactivation. CEN loops can stem from the chromosome arms and extend to the centromeres, originate from centromeres and extend to the chromosome arms, or both. If high transcription inhibits translocation of the cohesin complex, then the activation of GAL genes prior to Pds5 depletion should stop, or slow down, CEN loop formation. Consequently, the *GAL* gene locus should engage in loops with the same loci at which the CEN loops are maintained. To test these hypotheses, we monitored how active transcription of the *GAL7-10/1* locus interferes with the kinetics of CEN loop formation induced by controlled depletion of Pds5 using Hi-C and calibrated ChIP-seq. Pds5-AID cells expressing or not the *GAL4-ER-VP16* construct were arrested in G1 using the α factor. Estradiol was then added to both cultures, and the cells were released into the S phase and subsequently arrested in G2/M with nocodazole (Figures 6A and 6B). Auxin was added to the medium to induce degradation of Pds5-AID (Figures 6A and 6C). Following auxin addition, cells were sampled every 30 min for 2 h to monitor chromosome 3D organization with Hi-C (Figures 6D and S4B). Pds5-AID cells lacking the GAL4-ER-VP16 construct were used as controls. Pds5-AID depletion was achieved similarly in both conditions throughout the time course as assessed by the decrease of Smc3 acetylation (Figure 6C). At the same time, average contact frequencies as a function of genomic distance curves P(s) showed a progressive increase in long-range DNA contacts (Figure S4B). Normalized Hi-C maps, as well as 4C-like analysis using CEN2 as a genomic viewpoint, confirmed that Pds5 degradation in cells with silent *GAL7-10/1* engages *CEN2* in long-range, intra-arm contacts, resulting in CEN loops of increasing size (Figures 6D, 6E, and S4A). We verified using RNA Pol II ChIP-seq that transcription was not affected upon Pds5-AID depletion (Figure S4A). On the other hand, the pattern of *CEN2* contacts in the strain with *GAL7-10/1* activated prior to Pds5 degradation was very different. First, following Pds5 depletion, DNA loops bridging *CEN2* with loci across *GAL7-10/1* were strongly attenuated, while CEN loops bridging loci on the other chromosome arm of chr. 2 were unaffected (Figures 6E, 6F, and S4C). Large DNA loops increasing in size nevertheless gradually appeared along the arm, with the *GAL7-10/1* locus at their base instead of CEN2 (Figure 6F). This observation suggests that the expansion of CEN loops is halted by the highly expressed locus. In addition, this transcription-induced roadblock to loop expansion is semi-permissive, as loops between *CEN2* and distant loci, bridged across the *GAL7-10/1* locus, gradually accumulate over time, albeit to later time points than on the control kinetics (Figures 6E and S4C). Furthermore, the contacts made by DNA loops of the *GAL7-10/1* locus emanate from the same loci as those of regular CEN loops (Figures 6E and 6F). Here, too, a closer examination of cohesin deposition profiles showed that Pds5-AID degradation promotes their enrichment at the 3′ end of *GAL7*, *GAL10*, and *GAL1*, further supporting that active transcription tracks represent transient roadblocks to incoming cohesin, independently of the orientation of neighboring genes (Figure 6G). Altogether, these data demonstrate that local transcription acts as a semi-permissive barrier to cohesin-mediated loop expansion (model in Figure 7).

## Discussion

### The cohesin-dependent loop can be extended in the absence of RNA Pol II

The LE model proposes that cohesin captures small loops and progressively expands them by translocating onto DNA using the energy of ATP hydrolysis by Smc subunits. This model was strongly supported by single-molecule experiments showing that purified human or yeast cohesin can extrude DNA loops *in vitro* in an ATP-dependent manner. Despite these results, this model remains debated. A recent study revealed that mutant forms of cohesin unable to extrude a DNA loop *in vitro* are able to establish DNA loops in living cells, suggesting that cohesin-mediated DNA loops may not be established by the LE mechanism.^28^ The nature of the molecular motor of DNA loop expansion is also still debated. Another model proposes that DNA loop expansion is driven by RNA polymerase transcription, which pushes the cohesin holding the loop bases. The latter model is based on the fact that cohesin is mainly localized in the intergenic region, where transcription converges.^22^^,^^28^ However, our experiment demonstrates that cohesin can extend a DNA loop over a long distance on a chromosome lacking RNA Pol II (Figure 1). This indicates that RNA Pol II is not essential for cohesin-dependent DNA loop expansion. Expansion of the cohesin-dependent loops could, therefore, be driven by an active process stimulated by ATP hydrolysis of Smc subunits or by passive diffusion of cohesin rings along the DNA.

### Direct effect of transcription on chromatin long- and short-range folding

Both transcription and cohesin participate in genome folding, which in turn regulates DNA-related processes, including fine-tuned modulation of transcription itself.^46^^,^^47^ Here, we disambiguate this interplay by revealing the respective contributions of transcription and cohesin to chromatin organization. We show that active *GAL* promoters lead to the formation of TIDs^41^^,^^42^ and to the establishment of long-range DNA loops with neighboring transcribed loci, independently of individual SMC complexes (Figure 3). They may either result from the direct clustering of RNA Pol II complexes or transcription factors or from indirect contacts resulting from the relocation of the active regions to the same nuclear region, for instance, at the nuclear periphery.^48^^,^^49^^,^^50^ Enrichment in contacts between TIDs has been described in bacteria and mammals, although the nature of this phenomenon also remains unknown.^42^^,^^44^

### Impact of transcription on cohesin-mediated chromatin loops

Several studies have aimed at understanding how transcription influences the formation of cohesin-dependent chromatin loops.^12^^,^^25^^,^^29^^,^^30^^,^^31^ In the present study, we also precisely dissect the effect of transcription, through the proxy of GAL gene activation, on cohesin-mediated DNA loop formation, expansion, and maintenance. First, we show that transcription prevents or constrains the formation of cohesin-mediated DNA structures such as chromatin loops or stripes during the S phase (Figure 4). This suggests that transcription can interfere during the loading step of cohesin or on the loop expansion process by blocking the path to *cis*-translocating cohesin. To test this further, using a Pds5 mutant, we then performed a kinetics showing that the induced *GAL7-10/1* loci temporarily halt the expansion of centromeric-based DNA loops on the arm encompassing the GAL locus (Figure 6). This indicates that transcription acts as a semi-permissive roadblock to cohesin-mediated DNA loop expansion (Figure 6). Discrete stochastic bursts of transcription^51^ could account for how cohesin would bypass actively transcribed genes.

### A small proportion of cohesin may engage in DNA looping

Three pools of cohesin have been described so far, involved in (1) DNA looping, (2) sister-chromatid cohesion, and (3) topological entrapping of a single chromatid.^52^^,^^53^^,^^54^ As the cohesin ChIP-seq deposition profile does not discriminate between these populations, the relative proportions of cohesin involved in each pool, including the one involved in DNA looping, remain unknown. Our data show that a small quantity of cohesin is blocked at the 3′ ends of active *GAL7-10/1* genes (Figure 6G). This suggests that the overall amount of cohesin involved during the course of DNA looping may be relatively small. It is also possible that only a few cohesin rings are required to hold DNA loops. Alternatively, the dynamics of loop expanding cohesin might influence its capture by ChIP-seq, which relies on chromatin cross-linking. Highly dynamic cohesin binding could explain the observed low enrichment in ChIP-seq data.

### How does transcription slow down the cohesin-mediated DNA loop expansion process?

At least two models can explain how transcription positions DNA loops at the 3′ end of actively transcribed tracks. One possibility is that RNA Pol II dissociates translocating cohesin from the gene body, thereby limiting their position to the 3′ end of activated genes. Alternatively, RNA Pol II may collide with cohesin and push it toward the 3′ end of the gene. The latter scenario is the most likely, as evidenced by the effect of activating transcription of the *GAL2* locus in G2-arrested cells (Figure S3). Indeed, previous studies have revealed that RNA Pol II induces a shift of cohesin from the 5′ end to the 3′ end of activated *GAL2* even in the absence of *de novo* loading.^22^ Here, we showed that this cohesin displacement at *GAL2* is accompanied by a decrease in the corresponding loop size. Taken together, these data show that RNA Pol II is a mobile barrier that limits the size of DNA loops.

We revealed that DNA looping can be maintained at the 3′ end of co-oriented (*GAL7*) or divergent (*GAL10-1*) genes. Nevertheless, the base of most chromatin loops along budding yeast chromosomes is positioned between convergent genes, suggesting that in addition to RNA Pol II, whose effect can be bypassed, other mechanisms independent of a specific DNA sequence are required to maintain DNA loops at those specific loci. It is therefore likely that other factors, such as specific chromatin features, specific transcription factors, or topology that favors the stabilization of the loops, act in regions between convergent genes.

### Limitations of the study

In this study, we investigated the role of RNA Pol II in shaping the 3D organization of the genome, using *S. cerevisiae* as a model organism. Our findings reveal that transcription by RNA Pol II is not essential for the expansion of DNA loops mediated by the cohesin complex. Nonetheless, RNA Pol II can independently establish long-range DNA loops, and it also serves as a barrier that restricts the extension of cohesin-dependent loops. Further research will be necessary to elucidate the molecular mechanisms by which RNA Pol II promotes cohesin-independent loop formation and hinders cohesin-driven loop expansion. Additionally, it remains to be determined whether these mechanisms are conserved in other organisms.

## Resource availability

### Lead contact

Further information and requests for resources and reagents should be directed to and will be fulfilled by the lead contact, Frédéric Beckouët (frederic.beckouet@univ-tlse3.fr).

### Materials availability

All strains are available, without restriction, from the lead contact.

### Data and code availability

• The accession number for processed data for all figures is GEO: GSE232323.
• Raw sequences for all figures are accessible on the SRA database through the following accession number: SRA: PRJNA971611.
• Reference genomes for *S. cerevisiae* W303^55^ and *C. glabrata*^56^ are available here: http://www.candidagenome.org/download/sequence/C_glabrata_CBS138/current/.
• Programs involved in the study are listed below:
  HiCstuff (www.github.com/koszullab/hicstuff) v.3.0.1^37^ and Serpentine^37^ (www.github.com/koszullab/serpentine; v.0.1.3.)
  Bowtie2 (v.2.3.4.1, available online at http://bowtie-bio.sourceforge.net/bowtie2/)^57^
  Samtools (v.1.9, available online at http://www.htslib.org/download/)^58^
  Bedtools (v.2.29.1 available online at https://bedtools.readthedocs.io/en/latest/content/installation.html)^59^
  Cooler (v.0.8.7–0.8.11 available online at https://cooler.readthedocs.io/en/latest/)^60^
  bamCoverage (v.3.4.1) https://deeptools.readthedocs.io/en/develop/content/tools/bamCoverage.html)^61^
  Chromosight (www.github.com/koszullab/chromosight) v.1.6.3^37^
• The custom-made codes of the analysis are available online at https://github.com/axelcournac/RNAPII_loops and on Zenodo: https://doi.org/10.5281/zenodo.15681286.

## Acknowledgments

This research was supported by the 10.13039/501100000781European Research Council under the Horizon 2020 Program (ERC grant agreement 771813) to R.K. and 10.13039/501100001665Agence Nationale de la Recherche (ANR-22-CE12-0013-01) to F.B. and R.K. F.B., C.C., and A.L. received support from the 10.13039/501100004097Fondation ARC pour la Recherche sur le Cancer (ARCPJA2022060005240, ARCPJA2024060008359, and ARCPJA2023060006648) and the Comité de l’Occitanie de la Ligue Nationale contre le Cancer. N.B. was supported by the Ministère de l’Enseignement Supérieur and la Ligue Nationale contre le Cancer and Le comité départementale de la Ligue contre le Cancer de la Moselle. C.C. was supported by a Pasteur-Roux-Cantarini fellowship. A.L. was supported by the Agence Nationale de la Recherche (ANR-20-CE12-0016-01). L.C. was supported by the Ministère de l'Enseignement Supérieur (France). O.G. was supported by the Agence Nationale de la Recherche (ANR-23-CE12-0001). We thank all our colleagues from the teams Régulation spatiale des génomes and Organisation du noyau for helpful comments on the work, K. Nasmyth’s laboratory and B. Albert for strains, and K. Shirahige for the Smc3 antibody.

## Author contributions

Conceptualization, C.C., N.B., A.C., L.C., A.L., R.K., and F.B.; methodology, N.B., C.C., A.C., O.G., A.T., S.Q., L.C., A.L., H.M., R.K., and F.B.; investigation, N.B., C.C., L.C., R.K., A.L., and F.B.; formal analysis, C.C., N.B., H.M., A.C., and L.C.; data curation, N.B., C.C., A.C., A.L., L.C., F.B., and R.K.; visualization, N.B., A.L., C.C., A.C., F.B., L.C., and R.K.; writing, N.B., C.C., R.K., L.C., A.L., A.C., and F.B.; supervision, A.L., R.K., and F.B.; funding acquisition, O.G., C.C., A.L., F.B., and R.K.

## Declaration of interests

The authors declare no competing interests.

## STAR★Methods

### Key resources table

**Table:** 

REAGENT or RESOURCE	SOURCE	IDENTIFIER
**Antibodies**		

Anti-V5 monoclonal SV5-PK1 (Mouse)	BioRad	Cat# MCA1360; RRID: AB_322378
Anti-HA monoclonal 12CA5 (Mouse)	Sigma-Aldrich	Cat# 11583816001; RRID: AB_514505
Anti-pgk1 22C5D8 (Mouse)	Thermo Fisher Scientific	Cat# 459250; RRID: AB_2532235)
Anti-Sm3-K113Ac monoclonal H2 (Mouse)	K. Shirahige lab	N/A
Anti-mouse IgG, HRP conjugate	Promega	Cat#W4021; RRID: AB_430834
Anti-pol II (8WG16) (Mouse)	Merck Millipore	Cat# 05–952; RRID: AB_492629

**Chemicals, peptides, and recombinant proteins**		

Indole-3-acetic acid (IAA/Auxin)	Sigma	Cat# I3750-5G-A
Rapamycin	AbMole	Cat# M1768
RNase A	Fisher life Science	Cat# 10753721
α-factor peptid	Antibodies-online	Cat# ABIN399114
α-factor peptid	Proteogenix	Cat# WY-13
oestradiol	Sigma-Aldrich	Cat# E2758
Nocodazole	Sigma-Aldrich	Cat# M1404-10MG
Formaldehyde (Hi-C)	Sigma-Aldrich	Cat# F8775-2ML
Formaldehyde (ChIP)	VWR	Cat# 20909.290
trichloroacetic acid (TCA)	Sigma-Aldrich	Cat# T8657
Western ECL	Bio-Rad	Cat# 1705061
Propidium Iodide	Fisher Scientific	Cat# P3566
Glycine	Sigma-Aldrich	Cat# G7126-1KG
Mini-protean TGX stain-Free Gels	Bio-Rad	Cat# 2553068
ARIMA kit	Arima genomics	Cat# 510177
Colibri kit	Fisher Scientific	Cat# 16554311
AMPure XP beads	Beckman	Cat# A63882
cOmplete(TM), EDTA-free Protease In	Sigma-Aldrich	Cat# 5056489001
DTT	Sigma-Aldrich	Cat# D0632-5G
AEBSF	Euromedex	Cat# 50985-A
G dynabeads	Fisher Scientific	Cat# 10004D
Proteinase K	Sigma-Aldrich	Cat# 03115828001
Precellys Lysing kit VK05-0.5mL	Bertin technologies	Cat# P000934-LYSK0-A
microTUBE AFA Fiber Pre-Slit Snap-C	Covaris	Cat# 520045
Igepal	Sigma-Aldrich	Cat# I8896-50ML

**Deposited data**		

GEO accession number	This study	GSE232323
SRA database	This study	SRA: PRJNA971611

**Experimental models: Organisms/strains**		

Yeast strain used in this study (*S. cerevisiae* W303)		N/A
W303-1A (MATa)	Ralser et al.^62^	N/A
BEN15 (MATa, ura3:GAL4DBD-ER-Msn2-AD:URA3)	Bastié et al.^36^	N/A
FB220-1b (MATa ura3:GAL4DBD-ERMsn2-AD:URA3, his3:ADH1promoter-OsTIR1-9myc:HIS3)	This study	N/A
FB219-2a (MATa ura3:GAL4DBD-ERMsn2-AD:URA3, Scc1-PK3-aid:KanMX4,his3:ADH1promoter-OsTIR1-9myc:HIS3)	This study	N/A
FB242-8c (MATa ura3:GAL4DBD-ERMsn2-AD:URA3, his3:ADH1promoter-OsTIR1-9myc:HIS3, Smc5-AID:KanMX,Smc6-AID-9myc:hph)	This study	N/A
yCH001-9a (MATa ura3:GAL4DBD-ERMsn2-AD:URA3, his3:ADH1promoter-OsTIR1-9myc:HIS3, Smc2-AID-9myc:Nat)	This study	N/A
FB220-2a (MATa his3:ADH1promoter-OsTIR1-9myc:HIS3, Pds5-AID::KanMx)	This study	N/A
FB220-8c (MATa ura3:GAL4DBD-ERMsn2-AD:URA3,his3:ADH1promoter-OsTIR1-9myc:HIS3, Pds5-AID::KanMx)	This study	N/A
FB217-13C (MATa his3:ADH1promoter-OsTIR1-9myc:HIS3, SCC1-HA6:HIS3, ura3:GAL4DBD-ERMsn2-AD:URA3, Pds5-AID::KanMx)	This study	N/A
FB222-1c (MATa his3:ADH1promoter-OsTIR1-9myc:HIS3, SCC1-HA6:HIS3, Pds5-AID::KanMx)	This study	N/A
FB218-4a (MATa SCC1-HA6:HIS3)	This study	N/A
FB218-8D (MATa SCC1-HA6:HIS3,ura3:GAL4DBD-ER-Msn2-AD:URA3)		N/A
yNB30.2–14b (MATa SCC1-PK9:KanMX)	This study	N/A
PP6494 (MATa, pds5:HIS, pds5-101:LEU, tor1-1, fpr1:NAT,RPL13A-2XFKBP12:TRP1, RPB3-FRB-KanMX6, RPB1-FRBKanMX6)	This study	N/A
PP5617 (MATa, tor1-1, fpr1:NAT,RPL13A-2XFKBP12:TRP1, RPB3-FRB-KanMX6, RPB1-FRBKanMX6)	This study	N/A
PP5515 (MATa, tor1-1, fpr1:NAT, RPL13A-2XFKBP12:TRP1)	This study	N/A
*Candida Glabrata* KN23308 (Scc1-pk9:NatMX)	Hu et al.^43^	N/A
*Candida Glabrata* KN25532 (Scc1-HA3:NATMX)	Petela et al.^45^	N/A

**Software and algorithms**		

CytExpert 2.4 software a CyFlow ML Analyzer	Beckman	https://www.beckman.com/flow-cytometry/research-flow-cytometers/cytoflex/software
ChemiDoc Touch Imaging System: Image Lab 6.0.	BioRad	https://www.bio-rad.com/fr-fr/product/image-lab-software?ID=KRE6P5E8Z
HiCstuff version 3.0.1	http://www.github.com/koszullab/hicstuff	https://doi.org/10.5281/zenodo.8322591
Serpentine (version 0.1.3)	Matthey-Doret et al.^37^	www.github.com/koszullab/serpentine
Chromosight v1.3.1 36	Matthey-Doret et al.^37^	www.github.com/koszullab/chromosight
Samtools (version 1.9 available online)	Li et al.^58^	http://www.htslib.org/download/
Cooler (version 0.8.7–0.8.11 available online)	Abdennur and Mirny^60^	https://cooler.readthedocs.io/en/latest/
bamCoverage (version 3.4.1)	Ramirez et al.^61^	https://deeptools.readthedocs.io/en/develop/content/tools/bamCoverage.html
Bowtie2 (version 2.3.4.1 available online)	Langmead and Salzberg^57^	http://bowtie-bio.sourceforge.net/bowtie2/
Bedtools (version 2.29.1 available online at https://bedtools.readthedocs.io/en/latest/content/installation.html)	Quinlan and Hall^59^	https://github.co/arq5x/bedtools2
deepTools https://github.com/deeptools/deepTools.	Ramirez et al.^61^	https://deeptools.readthedocs.io/en/develop/content/about.html
Custom made code		https://doi.org/10.5281/zenodo.15681286 https://github.com/axelcournac/RNAPII_loops

### Experimental model and subject details

The yeast strains, derivative of *S.cerevisiae* W303 and *C.glabrata* are listed in the key resources table and in Table S1.

### Method details

#### Media culture conditions and synchronisation

All strains used in this study are derivative of W303 and are listed in the Table S1 “Strain list”. All strains were grown overnight at 30°C or 25°C in 150mL of suitable media supplemented with either 2% glucose or 2% galactose when indicated to attain 4,2 × 10ˆ8 cells. Yeast cells were synchronised in G1 by adding of α-factor (Antibodies-online, ABIN399114 or Proteogenix, WY-13) in the media every 30 min during 2h30 (1μg/mL final).

To arrest cells in metaphase, yeast cells were synchronised in G1 by adding of α-factor in the media every 30 min during 2h30 (1μg/mL final). After G1 arrest, cells were washed twice in fresh YP and released in rich medium (YP): 1%bacto peptone (Difco), 1%bacto yeast extract (Difco), supplemented with either 2% glucose or 2% galactose when indicated and containing Nocodazole (Sigma-Aldrich, M1404-10MG). Rapamycin (1μg/mL) (Interchim), Auxin (2mM final) (Sigma-Aldrich, I3750) and/or oestradiol (100nM final) (Sigma-Aldrich, E2758) was added to the media when indicated.

#### Flow cytometry

To verify cell-cycle arrest and synchronisation, 1mL of cells culture were fixed in ethanol 70% and stored overnight at −20°C. Pellet was incubated with 50mM Tris-HCl (pH 7,5) and 5μL RNase A (10mg/mL) overnight at 37°C. Cells were pelleted and re-suspended in 400μL of FACS buffer (1mg/mL propidium iodide (Fisher, P3566), Tris-HCl, NaCl, MgCl2) and incubated at 4°C. Cells were sonicated with 60% output for 10 s.

Flow cytometry was performed on a CytoFLEX S (Beckman Coulter) and data were analyzed using CytExpert 2.4 software, measuring 1000 events at a 30μL/min flow rate.

#### Protein extractions and acetylation assays

A pellet from 10mL of culture was frozen in liquid nitrogen and stored overnight at −20°C. Cell pellets were re-suspended in 100μL H2O and 20μL trichloroacetic acid (Sigma-Aldrich, T8657) (TCA). Then cells were broken by glass beads at 4°C and precipitated proteins were re-suspended in Laemmli buffer with 100mM DTT and Tris-HCl (pH 9,5). Proteins were extracted by cycles of 5 min heating at 80°C and 5min vortexing at 4°C. After centrifugation, extracted proteins were collected and froze at −20°C.

Eluates were analyzed by SDS-page followed by western blotting with antibodies.

(1) Mouse anti Smc3-K113Ac (Beckouet et al., 2010) (ab from K. Shirahige laboratory, clone H2) used at dilution 1:1000 for Western Blot
(2) Mouse anti-V5 tag (Bio-Rad, MCA1360) used at dilution 1:5000 for Western Blot
(3) Mouse anti-HA (Abcam, ab1424, 12CA5), used at dilution 1:1,000 for Western Blot
(4) Mouse anti-pgk1 (22C5D8) (Invitrogen, 459250) used at dilution 1:5000 for Western Blot
(5) Anti-mouse IgG, HRP conjugate (Promega, W4021) used at dilution 1:5000 for Western Blot

Blots were revealed using ChemiDoc Touch Imaging System: Image Lab 6.0.

The ratio (relative quantity of acSmc3/Pgk1) was calculated using the Volume tool of Image Lab: Background subtraction method: Local, Quantity regression method: Point to Point through origin.

#### Calibrated ChIP-sequencing

Cells were grown exponentially to OD600 = 0.5. In triplicates, 15 OD600 units of S. cerevisiae cells were mixed with 3 OD600 units of C. glabrata for Chip on cohesin or 0. 75 OD600 of C. glabrata for Chip on Pol II to a total volume of 45 mL and fixed with 4mL of fixative solution (50 mM Tris-HCl, pH 8.0; 100 mM NaCl; 0.5 mM EGTA; 1 mM EDTA; 30% (v/v) formaldehyde) for 30 min at room temperature (RT) with rotation. The fixative was quenched with 2mL of 2.5M glycine (RT, 5 min with rotation). The cells were then harvested by centrifugation at 3,500 rpm for 3 min and washed with ice-cold PBS. The cells were then resuspended in 300 mL of ChIP lysis buffer (50 mM HEPES KOH, pH 8.0; 140 mM NaCl; 1 mM EDTA; 1% (v/v) Triton X-100; 0.1% (w/v) sodium deoxycholate; 1 mM PMSF; 2X Complete protease inhibitor cocktail (Roche)) and transfer in tubes 2mL containing glass beads before mechanical cells lysis. The soluble fraction was isolated by centrifugation at 2,000 rpm for 3min then transferred to sonication tubes and samples were sonicated to produce sheared chromatin with a size range of 200-1,000bp. After sonication the samples were centrifuged at 13,200 rpm at 4°C for 20min and the supernatant was transferred into 700μL of ChIP lysis buffer. 80μL (27μL of each sample) of the supernatant was removed (termed ‘whole cell extract [WCE] sample’) and store at −80°C. 5ug of antibody (anti-HA or anti-Pol2 8WG16) was added to the remaining supernatant which is then incubated overnight at 4°C (wheel cold room). 50μL of protein G Dynabeads was then added and incubated at 4°C for 2h. Beads were washed 2 times with ChIP lysis buffer, 3 times with high salt ChIP lysis buffer (50mM HEPES-KOH, pH 8.0; 500 mM NaCl; 1 mM EDTA; 1% (v/v) Triton X-100; 0.1% (w/v) sodium deoxycholate; 1 mM PMSF), 2 times with ChIP wash buffer (10 mM Tris-HCl, pH 8.0; 0.25MLiCl; 0.5% NP-40; 0.5% sodium deoxycholate; 1mM EDTA; 1 mMPMSF) and 1 time with TE pH7.5. The immunoprecipitated chromatin was then eluted by incubation in 120μL TES buffer (50mM Tris-HCl, pH 8.0; 10 mM EDTA; 1% SDS) for 15min at 65°C and the supernatant is collected termed ‘IP sample’. The WCE samples were mixed with 40μL of TES3 buffer (50mM Tris-HCl, pH 8.0; 10mM EDTA; 3% SDS). ALL (IP and WCE) samples were de-cross-linked by incubation at 65°C overnight. RNA was degraded by incubation with 2μL RNase A (10 mg/mL) for 1h at 37°C. Proteins were removed by incubation with 10μL of proteinase K (18 mg/mL) for 2h at 65°C. DNA was purified by a phenol/Chloroform extraction. The triplicate IP samples were mixed in 1 tube and libraries for IP and WCE samples were prepared using Invitrogen TM Collibri TM PS DNA Library Prep Kit for Illumina and following manufacturer instructions. Paired-end sequencing on an Illumina NextSeq500 (2 × 35 bp) was performed.

#### Hi-C procedure and sequencing

Cell fixation with 3% formaldehyde (Sigma-Aldrich, Cat. F8775) was performed as described in Dauban et al. 2020 and Piazza et al. 2020.^18^^,^^63^ Quenching of formaldehyde with 300 mM glycine was performed at 4°C for 20 min. Hi-C experiments were performed with a Hi-C kit (Arima Genomics) with a double DpnII + HinfI restriction digestion following manufacturer instructions. Briefly, samples were permeabilised by sequentially adding lysis buffer (15min at 4C), conditioning solution (10min at 62C) and stop solution 2 (15min at 37C) to the samples. DNA was digested using a mix of buffer A, DpnII, and Hinf1 (45min at 37C followed by 20min at 65C). DNA was repaired and biotin was added by adding a mix of buffer B and enzyme B for 45min at room temperature. DNA was re-ligated by adding a mix of buffer C and enzyme C during 15min at room temperature. Samples were then digested by protease and de-crosslinked by adding a mix of buffer D, enzyme D and buffer E during 30 min à 55°C followed by 90 min at 68°C. Samples were purified using AMPure XP beads (Beckman A63882), recovered in 120ul H2O and sonicated using Covaris (DNA 300bp). Preparation of the samples for paired-end sequencing on an Illumina NextSeq500 (2 × 35 bp) was performed using Invitrogen TM Collibri TM PS DNA Library Prep Kit for Illumina and following manufacturer instructions.

### Quantification and statistical analysis

#### Processing of the reads and generation of calibrated ChIP-seq profiles

For analysis, Bowtie2 was used for two rounds of alignments, first on *C. glabrata* (CBS138) and then on *S. cerevisiae* allowing the generation of an alignment of IP and WCE that exclusively mapped on *S. cerevisiae* (an vice-versa for *C. glabrata*). The obtained SAM file was converted into a BAM file, sorted and indexed using Samtools. ChIP-seq profiles were then normalised by the number of million sequences and converted into BigWig using bamCoverage. Profiles were multiplied by the ORi factor (WCEglabrataIPcerevisiae/WCEcerevisiaeIPglabrata, in which WCE glabrata and IPglabrata correspond to the number of paired reads that mapped uniquely on *C. glabrata* genome and same for *S. cerevisiae* reads) using Integrated Genome Browser.

Final number of reads and corresponding experiments are listed in Table S2.

#### Processing of the reads, computation of contact matrices, and generation of contact maps

Reads were aligned and the contact data processed using Hicstuff, available on Github (https://github.com/koszullab/hicstuff). Briefly, pairs of reads were aligned iteratively and independently using Bowtie2 in its most sensitive mode against the *S. cerevisiae* W303 reference genome. Each uniquely mapped read was assigned to a restriction fragment. Quantification of pairwise contacts between restriction fragments was performed with default parameters: uncuts, loops and circularization events were filtered as described previously.^36^^,^^57^

PCR duplicates (defined as paired reads mapping at exactly the same position) were discarded. Contact maps from independent replicates were generated with the “view” function of Hicstuff, merged and normalized using the merge and balance functions of Cooler. Bins were set at 1, exp0.2 transformed, and rendered.

Final number of reads and corresponding experiments are listed in Table S2.

#### Computation of the contact probability as a function of genomic distance

Computation of the contact probability as a function of genomic distance P(s) and its derivative have been determined using the “distance law” function of Hicstuff with default parameters, averaging the contact data of entire chromosome arms. P(s) from two independent replicates were determined and plotted.

#### Generation of the ratio maps using serpentine

Ratio maps were generated with Serpentine.^37^ Briefly, comparison between pairs of 1-kb contact maps was performed using the default threshold parameters and the detrending constant was determined automatically by Serpentine for each comparison.

#### Virtual 4C profiles

4C-like profiles were generated on 2kb binned contact maps for a region of interest using an 8kb window as bait.

#### Loop spectrum using chromosight “quantify” mode

The Loop Spectrums were computed as explained in.^37^ First, peaks of RNA Pol II were extracted from Rpb3 ChIP-seq profiles generated during in log phase^40^ with homemade python codes. Enrichment in contacts (i.e., “loop scores”) between all pairs of loci enriched for a peak in Rpb3 were computed using Chromosight, and the average loop scores were computed for different sizes of loops on Micro-C contact data from,^40^ (SRR13736654). A locally weighted scatterplot smoothing (lowess) was then applied using scikit-misc package.

The pileups plots of either windows centered on RNA Po lII peaks along the diagonal, or of pairs of windows centered on distant RNA Pol II peaks (60 kb–460 kb), were computed for five different groups of RNA peaks sorted by the transcription level (with ChIP-seq of Rpb3 data of^40^). The different groups of transcription levels were determined by computing the distribution of ChIP-Seq intensities for all 2 kb bins of the *S. cerevisiae* genome. Then the five groups were defined with the following limits: [0:0,5],[0,5:1,0],[1:1,25],[1,25:1,5] and [1,5:15] corresponding to regions from low to high RNA Pol II levels, respectively.

Pileup plots during the mitotic cell cycle were generated with 2 different group of pairs: pairs of cohesin peaks between 10 kb and 50 kb and pairs of Pol II peaks between 60 kb and 460 kb with the contact data from,^19^ (SRR11893107). Pileup plots for SMC mutants were generated with the same group of pairs of cohesin peaks between 10 kb and 50 kb.

## Supplemental information

Document S1. Figures S1–S4 and Table S1

Table S2. Sequencing datasets used in this study

Document S2. Article plus supplemental information
