# Supplementary material for: RNA Pol II-based regulations of chromosome folding
Source: Cell Genom. 2025 Aug 8;5(10):100970. doi: 10.1016/j.xgen.2025.100970 (PMC12790994; doi:10.1016/j.xgen.2025.100970)
Supplement: Document S1. Figures S1–S4 and Table S1 [file mmc1.pdf]

Cell Genomics, Volume *5*

Supplemental information

RNA Pol II-based regulations of chromosome folding

Christophe Chapard, Nathalie Bastié, Axel Cournac, Laura Chaptal, Henri Mboumba, Sophie Queille, Agnes Thierry, Olivier Gadal, Armelle Lengronne, Romain Koszul, and Frédéric Beckouët

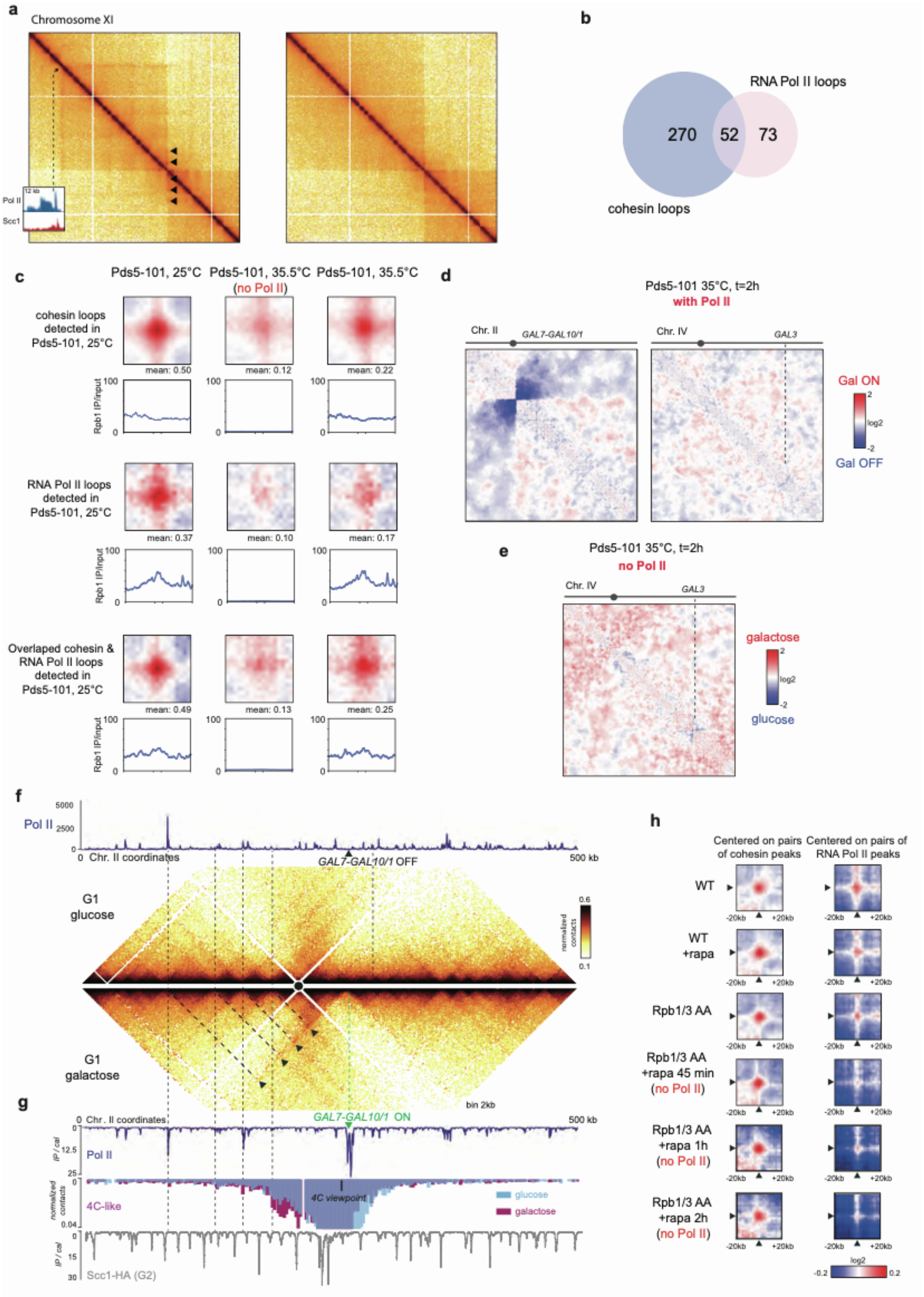

Figure S1. Pol II transcription influences genome organization; related to figures 1 and 2.

A) Hi-C contact maps (bin: 2 kb) of chromosome XI for *pds5-101* cells (PP6494) at restrictive temperature (35°C). Black arrowheads point at CEN loops. A dotted, arrowhead black line points at the basis of a stripe-like structure, accompanied by a magnification centered on the basis and highlighting the corresponding local Pol II and cohesin distribution assessed by ChIP-seq experiment. B) Proportion of loops anchored by Scc1 peaks, Pol II peaks, or both (maps Fig1e). C) Mean profile heatmap of cohesin and PolII loops using the 'quantify' mode of Chromosight[1] in presence or absence of transcription and/or active Pds5 (maps in Fig. 1**e**). Under the heatmap, pile-up of 15 kb windows of calibrated Chip-seq tracks centered on Pol II peaks are represented. Loops were called by Chromosight on the *pds5-101* Hi-C map at permissive temperature (25°C) and computed using the pairs of peaks of cohesin or Pol II for the datasets. D) Ratio of contact maps obtained from G2/M arrested cells cultured with (GAL ON, strain FB220-1b) or without oestradiol-(GAL OFF, strain W303-1A) and processed through Serpentine[1] as described in the material and methods: chromosomes II (left), IV (right). E) Ratio of contact maps of chromosome IV obtained from G2 cells (W303-1A) cultured with glucose or galactose. F) Hi-C contact maps (bin: 2kb), and corresponding Pol II ChIP-seq profiles, of a section of chromosome II containing the *GAL7-10/1* locus in cells (W303-1A) synchronized in G1 in either glucose (top) of galactose (bottom). Loops with the *GAL7-10/1* locus as one basis are indicated with black triangles. For each loop, the coordinate of the other basis is identified following the blue, then dark, dotted lines.

G) Upper panel: A virtual 4C plot with a viewpoint (anchor) just upstream the GAL7-10/1 locus of cells in either glucose (pink) or galactose (light blue) is shown below Pol II Chip-seq profile in galactose (W303-1A). Lower panel: Lower panel: A cohesin (Scc1) ChIP-seq profile of G2 arrested cells (yNB30.2-14b) is shown in grey.

H) Pile up plot for RNA Pol II enriched regions over long distances (60 kb – 460 kb) and for cohesin loops for WT (PP5515) and Pol II depleted cells (PP5617).

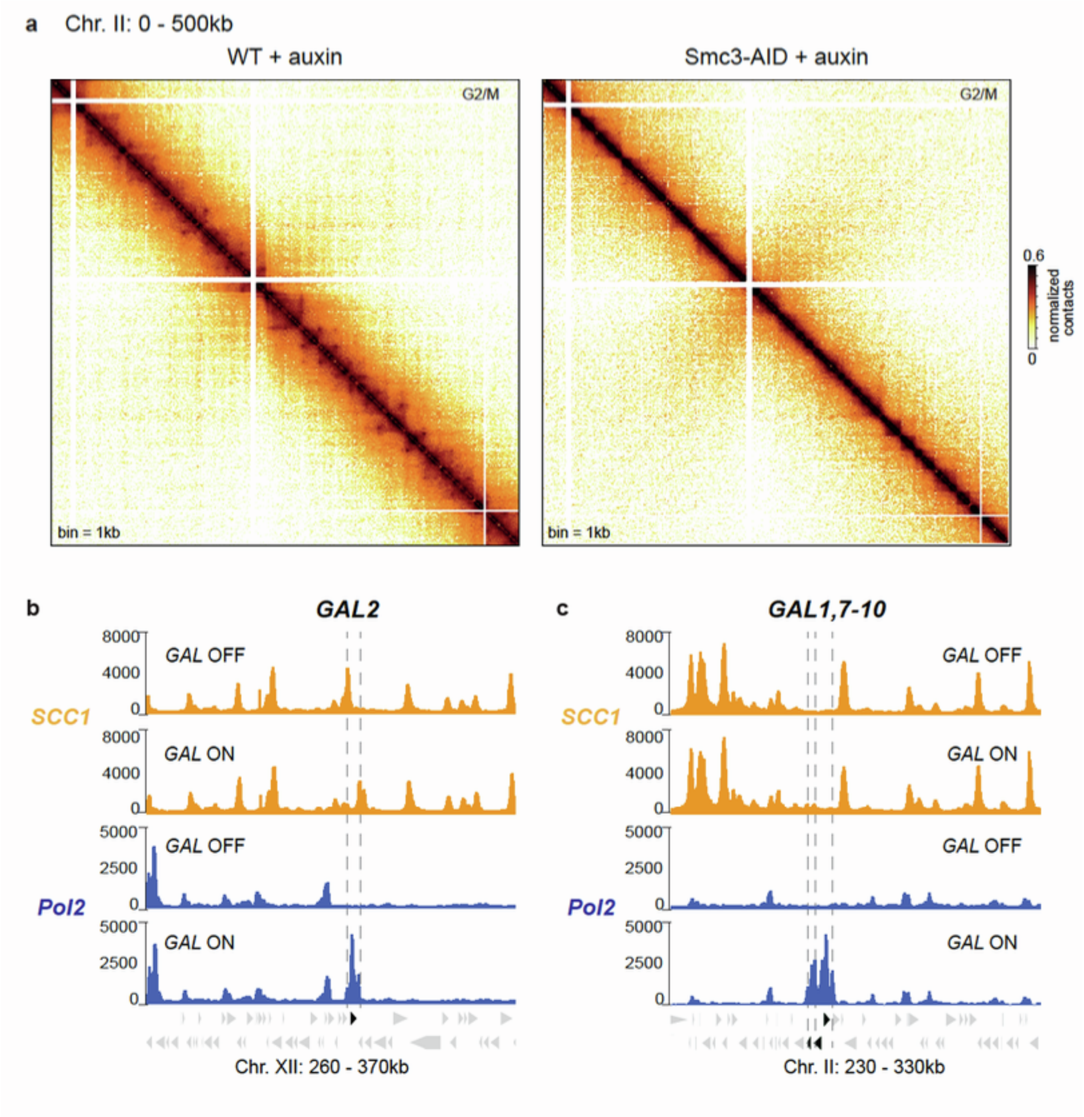

Figure S2. Pol II transcription interferes with the establishment of cohesin-dependent structures; related to Figure 4.

a) Hi-C contacts maps for W303-1A and FB134-16c cells showing effect of Smc3 inactivation (Smc3-AID + auxin) on chromosome organization.

b) Pol II (blue) and Scc1 (orange) ChIP-seq profiles of a region of chr. XII containing the *GAL2* locus (Strains FB218-4a and FB218-8D). Cells are synchronized in metaphase, without (GAL OFF) or with (GAL ON) oestradiol-induced activation of *GAL2*.

c) Pol II (blue) and Scc1 (orange) ChIP-seq profiles of a region of chr. II containing the *GAL7-10/1* locus (Strains FB218-4a and FB218-8D). Cells are synchronized in metaphase, without (GAL OFF) or with (GAL ON) oestradiol-induced activation of *GAL7-10/1*.

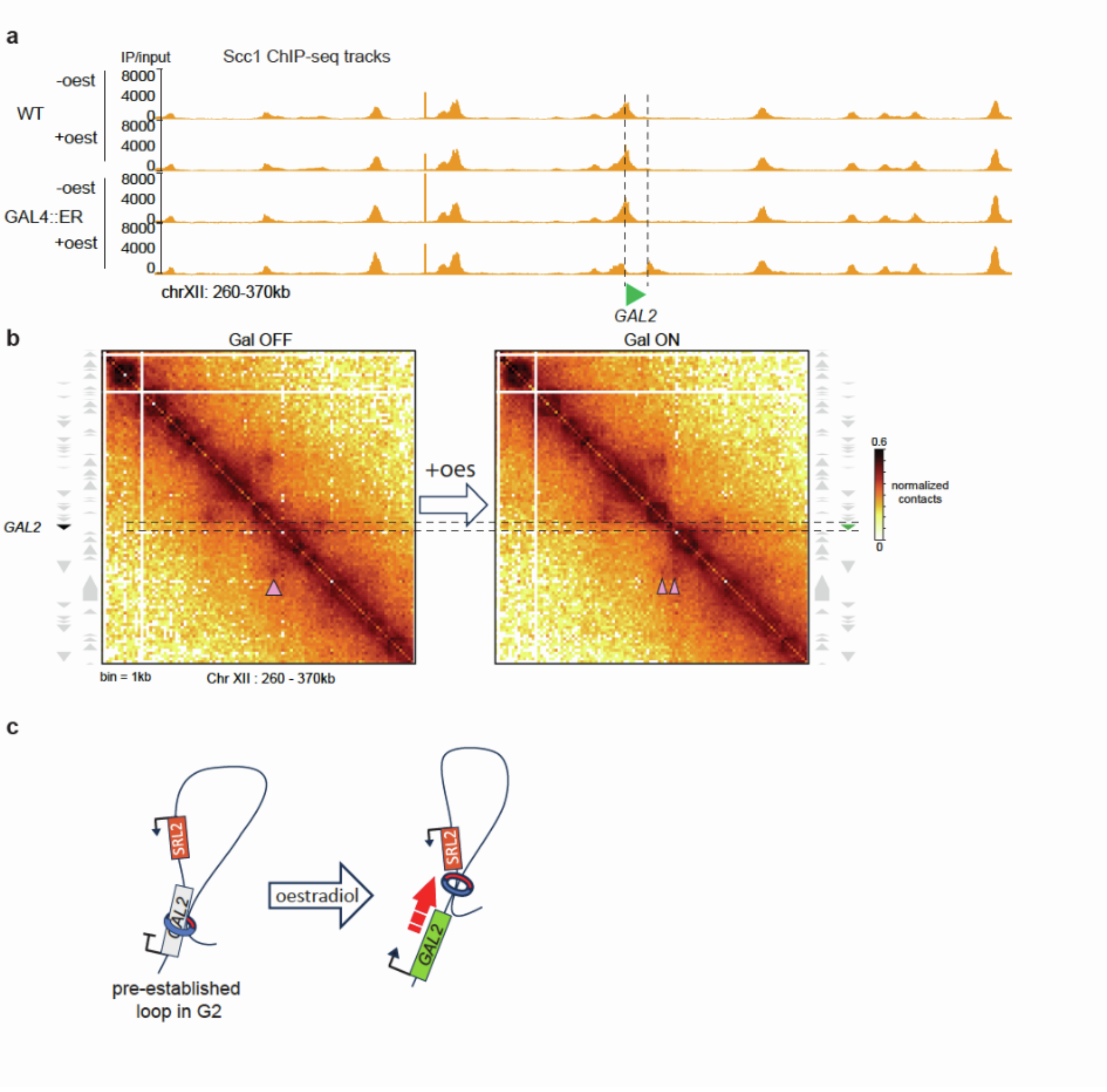

Figure S3. Effect of Pol II on the maintenance of cohesin-dependent structures; related to Figure 5.

a) Scc1 ChIP-seq of a section of chromosome XII in cells for the strains FB218-4a and FB218-8d cells arrested in G2 before (1st and 3rd tracks) and after adding oestradiol (2sd and 4th tracks). Dotted lines represent 5' and 3' of Gal2 gene. b) Contact maps (bin 1kb) of a section

of chromosome XII in BEN15 cells arrested in G2 in absence (left) or in presence (right) of GAL activation. Dotted lines represent 5’ and 3’ of Gal2 gene. The pink arrows indicate changes in DNA loop positioning. c) Illustration explaining effect of transcription on the maintenance of DNA loop in G2/M when the bases of DNA loop are placed within the ORF of induced gene.

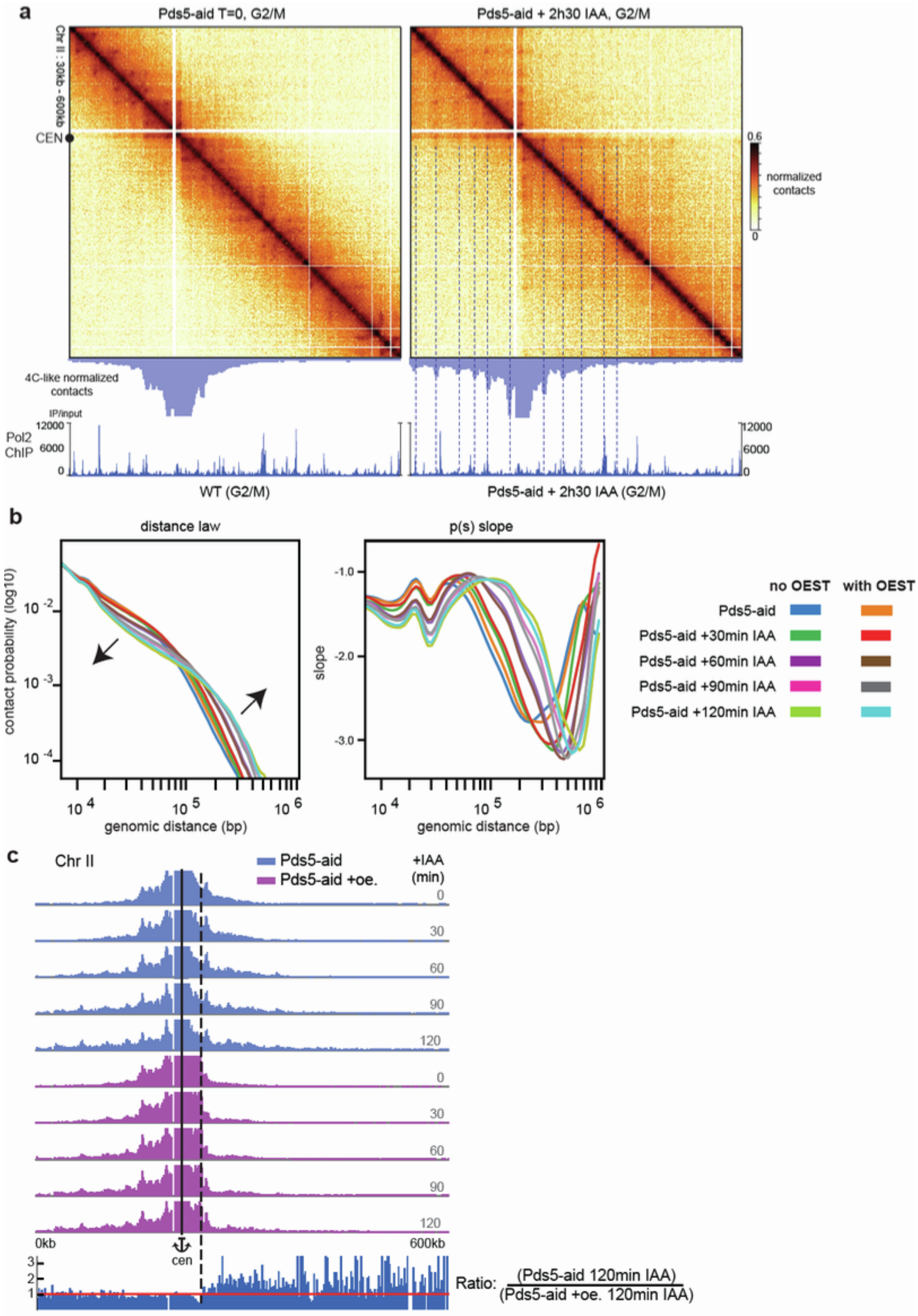

Figure S4. Pol II transcription slowdowns the expansion of cohesin-dependent loops; related to Figure 6.

a) Hi-C contact maps for FB220-2a cells (bin: 2kb), and corresponding Pol II ChIP-seq profiles, of a section of chromosome II containing the *GAL7-10/1 locus* in presence (left) or after

depletion of Pds5 mediated by IAA (right). A virtual 4C plot with a viewpoint centred on the centromere is shown for each map. b) Contact frequency curves P(s), representing average contact frequency as a function of genomic separation (bp), and their respective derivative curves throughout Pds5 depletion from 0min to 120min after IAA addition (strains: FB220-2a, FB220-8c). c) Magnifications of the 4C-like profiles using CEN as an anchor along chr. II in absence (Top blue) or in presence (Bottom pink) of GAL genes induction, following a time-coursed IAA-mediated Pds5 depletion. A ratio of the 4C-like profiles at 120 min after IAA-mediated Pds5 depletion, is provided showing fold enrichment of contacts with the CEN in absence versus presence of oestradiol induced GAL activation.

Supplementary Table 1 (related to all Figures)

Yeast strains

Strain name	Background	genotype	Figure	Ref
W303-1A	W303	MATa	Fig 2, Fig 4, Fig 5, sup Fig 1, sup Fig 2	[2]
BEN15	W303	MATa ura3:GAL4DBD-ER-Msn2-AD:URA3	Fig2, Fig 5, sup Fig 3	[3]
FB220-1b	W303	MATa ura3:GAL4DBD-ER-Msn2-AD:URA3, his3::ADH1promoter-OsTIR1-9myc::HIS3	Fig 3, Fig 4, sup fig 1	This study
FB219-2a	W303	MATa ura3:GAL4DBD-ER-Msn2-AD:URA3, Scc1-PK3-aid::KanMX4, his3::ADH1promoter-OsTIR1-9myc::HIS3	Fig 3	This study
FB242-8c	W303	MATa ura3:GAL4DBD-ER-Msn2-AD:URA3, his3::ADH1promoter-OsTIR1-9myc::HIS3, Smc5-AID:KanMX, Smc6-AID-9myc:hph	Fig 3	This study
yCH001-9a	W303	MATa ura3:GAL4DBD-ER-Msn2-AD:URA3, his3::ADH1promoter-	Fig 3	This study

		OsTIR1-9myc::HIS3, Smc2-AID-9myc:Nat		
FB220-2a	W303	MATa his3::ADH1promoter-OsTIR1-9myc::HIS3, Pds5-AID::KanMx	Fig 6, sup Fig 4	[4]
FB220-8c	W303	MATa ura3:GAL4DBD-ER-Msn2-AD:URA3, his3::ADH1promoter-OsTIR1-9myc::HIS3, Pds5-AID::KanMx	Fig 6, sup Fig 4, sup Fig 5	[4]
FB217-13C	W303	MATa his3::ADH1promoter-OsTIR1-9myc::HIS3, SCC1-HA6::HIS3, ura3:GAL4DBD-ER-Msn2-AD:URA3, Pds5-AID::KanMx	Fig 6	This study
FB222-1c	W303	MATa his3::ADH1promoter-OsTIR1-9myc::HIS3, SCC1-HA6::HIS3, Pds5-AID::KanMx	Fig 6	This study
FB218-4a	W303	MATa SCC1-HA6::HIS3	Fig 2, Fig 4, Fig 5, sup Fig 2, sup Fig 3	This study
FB218-8D	W303	MATa SCC1-HA6::HIS3, ura3:GAL4DBD-ER-Msn2-AD:URA3	Fig 2, Fig 4, Fig 5, sup Fig 2, sup Fig 3	This study
KN25532	Candida Glabrata	Scc1-HA3::NatMX	Fig 4, Fig 5, Fig 6, sup Fig 2, sup Fig 3	[5]
KN23308	Candida Glabrata	Scc1-pk9::NatMX	Fig 2, Sup Fig 1	[6]
yNB30.2-14b	W303	MATa SCC1-PK9::KanMX	Fig 2, Sup Fig 1	This study
PP6494	W303	MATa, pds5::HIS, pds5-101::LEU, tor1-1, fpr1::NAT, RPL13A-2XFKBP12::TRP1, RPB3-FRB-KanMX6, RPB1-FRB-KanMX6	Fig 1	This study
PP5617	W303	MATa, tor1-1, fpr1::NAT, RPL13A-2XFKBP12::TRP1, RPB3-FRB-KanMX6, RPB1-FRB-KanMX6	Sup Fig1	This study
PP5515	W303	MATa, tor1-1, fpr1::NAT, RPL13A-2XFKBP12::TRP1	Sup Fig1 (control)	This study
FB134-16c	W303	MATa, his3::ADH1promoter-OsTIR1-9myc::HIS3, SMC3-3sIAA::ADE2, TRP1:Metp:CDC20	Sup Fig2	[4]

References

1. Matthey-Doret, C., Baudry, L., Breuer, A., Montagne, R., Guiglielmoni, N., Scolari, V., Jean, E., Campeas, A., Chanut, P.H., Oriol, E., et al. (2020). Computer vision for pattern detection in chromosome contact maps. Nat. Commun. *11*, 5795. https://doi.org/10.1038/s41467-020-19562-7.

2. Ralser, M., Kuhl, H., Ralser, M., Werber, M., Lehrach, H., Breitenbach, M., and Timmermann, B. (2012). The Saccharomyces cerevisiae W303-K6001 cross-platform

genome sequence: insights into ancestry and physiology of a laboratory mutt. Open Biol. *2*, 120093. https://doi.org/10.1098/rsob.120093.

3. Pincus, D., Aranda-Díaz, A., Zuleta, I.A., Walter, P., and El-Samad, H. (2014). Delayed Ras/PKA signaling augments the unfolded protein response. Proc. Natl. Acad. Sci. U. S. A. *111*, 14800–14805. https://doi.org/10.1073/pnas.1409588111.

4. Dauban, L., Montagne, R., Thierry, A., Lazar-Stefanita, L., Bastié, N., Gadal, O., Cournac, A., Koszul, R., and Beckouët, F. (2020). Regulation of Cohesin-Mediated Chromosome Folding by Eco1 and Other Partners. Mol. Cell *77*, 1279-1293.e4. https://doi.org/10.1016/j.molcel.2020.01.019.

5. Petela, N.J., Gligoris, T.G., Metson, J., Lee, B.-G., Voulgaris, M., Hu, B., Kikuchi, S., Chapard, C., Chen, W., Rajendra, E., et al. (2018). Scc2 Is a Potent Activator of Cohesin's ATPase that Promotes Loading by Binding Scc1 without Pds5. Mol. Cell *70*, 1134-1148.e7. https://doi.org/10.1016/j.molcel.2018.05.022.

6. Hu, B., Petela, N., Kurze, A., Chan, K.-L., Chapard, C., and Nasmyth, K. (2015). Biological chromodynamics: a general method for measuring protein occupancy across the genome by calibrating ChIP-seq. Nucleic Acids Res. *43*, e132. https://doi.org/10.1093/nar/gkv670.
